# Evaluation of neurological effects of cerium dioxide nanoparticles doped with different amounts of zirconium following inhalation exposure in mouse models of Alzheimer's and vascular disease

**DOI:** 10.1016/j.neuint.2020.104755

**Published:** 2020-09

**Authors:** Tina Wahle, Adriana Sofranko, Susan Dekkers, Mark R. Miller, Harm J. Heusinkveld, Catrin Albrecht, Flemming R. Cassee, Roel P.F. Schins

**Affiliations:** a IUF - Leibniz Research Institute for Environmental Medicine, Düsseldorf, Germany; b National Institute for Public Health and the Environment, Bilthoven, the Netherlands; c Centre for Cardiovascular Science & Centre for Inflammation Research, University of Edinburgh, Edinburgh, United Kingdom; d Institute for Risk Assessment Sciences, Faculty of Science, Utrecht University, Utrecht, the Netherlands

**Keywords:** Cerium dioxide, Nanoparticles, Inhalation, Alzheimer's disease, Amyloid-β

## Abstract

Increasing evidence from toxicological and epidemiological studies indicates that the brain is an important target for ambient (ultrafine) particles. Disturbance of redox-homeostasis and inflammation in the brain are proposed as possible mechanisms that can contribute to neurotoxic and neurodegenerative effects. Whether and how engineered nanoparticles (NPs) may cause neurotoxicity and promote neurodegenerative diseases such as Alzheimer's disease (AD) is largely unstudied.

We have assessed the neurological effects of subacute inhalation exposures (4 mg/m^3^ for 3 h/day, 5 days/week for 4 weeks) to cerium dioxide (CeO_2_) NPs doped with different amounts of zirconium (Zr, 0%, 27% and 78%), to address the influence of particle redox-activity in the 5xFAD transgenic mouse model of AD. Four weeks post-exposure, effects on behaviour were evaluated and brain tissues were analysed for amyloid-β plaque formation and reactive microglia (Iba-1 staining). Behaviour was also evaluated in concurrently exposed non-transgenic C57BL/6J littermates, as well as in Western diet-fed apolipoprotein E-deficient (ApoE^-/-^) mice as a model of vascular disease. Markers of inflammation and oxidative stress were evaluated in brain cortex.

The brains of the NP-exposed 5xFAD mice revealed no accelerated amyloid-β plaque formation. No significant treatment-related behaviour impairments were observed in the healthy C57BL/6J mice. In the 5xFAD and ApoE^-/-^ models, the NP inhalation exposures did not affect the alternation score in the X-maze indicating absence of spatial working memory deficits. However, following inhalation exposure to the 78% Zr-doped CeO_2_ NPs changes in forced motor performance (string suspension) and exploratory motor activity (X-maze) were observed in ApoE^-/-^ and 5xFAD mice, respectively. Exposure to the 78% doped NPs also caused increased cortical expression of glial fibrillary acidic protein (GFAP) in the C57BL/6J mice. No significant treatment-related changes neuroinflammation and oxidative stress were observed in the 5xFAD and ApoE^-/-^ mice.

Our study findings reveal that subacute inhalation exposure to CeO_2_ NPs does not accelerate the AD-like phenotype of the 5xFAD model. Further investigation is warranted to unravel whether the redox-activity dependent effects on motor activity as observed in the mouse models of AD and vascular disease result from specific neurotoxic effects of these NPs.

## Highlights

• 4-week mouse inhalation study with 0%, 27% and 78% Zr-doped CeO_2_ nanoparticles.
• No acceleration of Alzheimer-related features in 5xFAD mouse model.
• Motor performance changes in 78% Zr-doped CeO_2_ exposed ApoE^-/-^ and 5xFAD mice.
• Increased GFAP levels in 78% Zr-doped CeO_2_ exposed C57BL/6J mice.

## 1 Introduction

Several research groups have postulated that ultrafine air pollution particles are an important environmental risk factor for neurotoxicity and, more specifically, may potentiate the risk of neurodegenerative disorders, like Alzheimer's Disease (AD) (reviewed in (Heusinkveld et al. 2016). In relation to this, concerns have been raised about the potential neurotoxic and neurodegenerative effects of engineered nanoparticles (NPs). However, despite great progress in nanotechnologies, comparatively little is known to date on the potential adverse effects that exposure to manufactured NPs may have on the human brain, including the potential induction of pathways leading to neurodegeneration (Cupaioli et al. 2014). Indeed, NPs can enter the human body through several routes, e.g. via inhalation, absorption from the digestive tract, or following injection into the blood in nanomedical applications. With regard to potential adverse impacts on the brain, uptake and retrograde axonal transport of NPs via the olfactory nerve has been demonstrated in rodent inhalation studies (Oberdorster et al. 2004; Elder et al. 2006; Elder and Oberdorster, 2006). Besides, NPs may reach the central nervous system via the blood–brain barrier (BBB), where they have been suspected to impair several molecular pathways and contribute to neurodegeneration (Iqbal et al. 2013; Cupaioli et al. 2014). The ability to generate reactive oxygen species and associated inflammation is considered one of the key mechanisms of nanomaterials' toxicity to the respiratory tract and cardiovascular system (Unfried et al. 2008; Miller et al., 2012; Stone et al. 2017) and thus could also play a major role in their neurotoxic and neurodegenerative effects. Indeed, oxidative stress and neuroinflammation have long been recognised in neurotoxicity and neurodegenerative diseases including AD (Heneka et al. 2015; Zhao and Zhao, 2013).

Among the various types of NPs, cerium oxide NPs (CeO_2_ NPs) have been subjected to various toxicological investigations in relation to inhalation exposure (Cassee et al. 2011; Demokritou et al. 2013). CeO_2_ NPs are widely used as catalysts in industrial applications. They are used as additive to diesel fuels in order to reduce the amount of emitted pollutants after their combustion. Because of their radical-scavenging properties, CeO_2_ NPs have gained strong interest in the field of nanomedicine (reviewed in (Das et al. 2013)). The antioxidant properties of CeO_2_ NPs are accomplished through its ability to switch from the 3+ to the 4+ valence state (Hirst et al. 2009). It has been shown that the antioxidant efficacy of CeO_2_ NPs can be affected by incorporation of zirconium (Zr) in the CeO_2_ lattice (Tsai et al. 2008). However, whilst research has been devoted since many years to elaborate on neuroprotective and potential anti-neurodegenerative effects of CeO_2_ (Singh et al., 2007), adverse effects on the brain should also be considered for this type of nanoparticles as indicated e.g. from intravenous application studies in rats (Hardas et al. 2010, 2014) and *in vitro* neuronal activity experiments with primary rat cortex cultures (Strickland et al. 2016).

Given that free radicals play a prominent role in the pathology of many neurological diseases, we explored the neurotoxicity of CeO_2_ NPs doped with varying amounts of Zr following inhalation exposure in three different mouse models, i.e. C57BL/6J, 5xFAD and ApoE^-/-^ mice. The 5xFAD transgenic mice were used in this study as a model for AD. The 5xFAD mouse model was used in a previous study, in which we have demonstrated that inhalation exposure to diesel engine exhaust results in an accelerated formation of Aβ-plaques as well as motor function impairment (Hullmann et al. 2017). Diesel engine exhaust represents a major source of unintentionally generated NPs in most urban environments and therefore supports the selection of the 5xFAD model for the investigation of the neurological effects of engineered NPs after inhalation. The nontransgenic littermate controls of the 5xFAD mice (C57BL/6J background) were used as a healthy mouse model. Finally, apolipoprotein E-deficient (ApoE^-/-^) mice, subjected to a high-fat diet, were included in the present study. ApoE^-/-^ mice represent a well-established model for the study of atherosclerosis, a disease characterized by the build-up of lipid- and inflammatory cell-rich plaques within arteries, which underlies the majority of cardiovascular diseases (Cassee et al. 2012; Miller et al. 2013). Since this ApoE deficiency compromises the blood brain barrier (Methia et al. 2001) this model could also be useful to study the susceptibility to NP-induced neurological effects. The adverse cardiovascular effects of diesel exhaust particles as well as specific types of engineered NPs have been clearly demonstrated in ApoE^-/-^ mice in several studies (Hansen et al. 2007; Kang et al. 2011; Miller et al. 2013). Interestingly, a comparative inhalation study with engine exhausts generated using fuels with or without added CeO_2_ NPs in ApoE^-/-^ mice revealed differences in atherosclerotic plaque formation but also in pro-inflammatory responses in (sub)cortical brain regions (Cassee et al. 2012; Lung et al. 2014), which could reflect a direct effect of these redox active NPs on the central nervous system.

The aim of the current study was to evaluate the potential neurotoxic and neurodegenerative effects of CeO_2_ NPs in mice following a four-week inhalation exposure and to assess the influence of redox activity by the concurrent evaluation of CeO_2_ NPs with different Zr-doping grades. The investigations formed part of a large study conducted in to explore the (patho)physiological effects of NP exposure on multiple organ systems in various mouse models (Dekkers et al. 2017, 2018).

## 2 Methods

### 2.1 Animals

In this study, three different mouse models were used. The 5xFAD transgenic mice were used as a model for AD. Only the female mice were used for the study in view of the reported sex-specific differences in age- and treatment related Aβ development (Devi et al. 2010). The 5xFAD mice overexpress the 695 amino acid isoform of the human amyloid precursor protein (APP695) carrying Swedish (K670N), London (V717I) and Florida (I716V) mutations as well as the human PS1 (M146L; L286V) mutations (Oakley et al. 2006; Ohno et al. 2004). The mice develop a specific phenotype that includes high APP expression levels, amyloid deposition (beginning at two months of age) and memory impairments and motor deficits (Oakley et al. 2006; Jawhar et al. 2012). The breeding was performed by mating heterozygote transgenic founders with C57BL/6J wild-type mice. The nontransgenic female littermates were used as model of healthy mice in this study. The 5xFAD and C57BL/6J mice originated from Jackson Laboratories. For the study, female 5xFAD mice (n = 64) and female cross bred C57BL/6J littermates (n = 40) were used at the age of 8–11 weeks. As a third model, ApoE^-/-^ mice were used. Female ApoE^-/-^ mice (n = 32) were obtained from Taconic, Denmark at age 10–12 weeks at the beginning of the study. The four-week inhalation exposure protocol in the ApoE^-/-^ mice was integrated into an 8-week high-fat (Western diet) feeding regime (Purified Diet Western 4021.06, ABdiets, Woerden, The Netherlands), which has been shown to generate complex atherosclerotic plaques with many of the hallmarks of the human disease in specific arterial locations (Cassee et al. 2012; Miller et al. 2013; Dekkers et al. 2017). All mice were barrier maintained and housed in a single room in macrolon cages. Temperature and relative humidity were controlled at 22 ± 2 °C and at 40–70%, respectively. Lighting was artificial with a sequence of 12 h light (during daytime) and 12 h dark (at night). Feed and drinking water were provided *ad libitum* from the arrival of the mice until the end of the study, except during exposure. The study was conducted at Intravacc (Bilthoven, The Netherlands) under a protocol approved by the Ethics Committee for Animal Experiments of the RIVM and performed according to applicable national and EU regulations.

### 2.2 Inhalation study design

The mice were exposed via nose only inhalation to CeO_2_ NPs with varying amounts of Zr-doping (0%, 27% or 78% Zr) or clean air, respectively, over a four-week period (4 mg/m^3^ for 3 h/day, 5 days/week). The number of animals per treatment group designed for the present study was n = 10 for the C57BL/6J mice, n = 16 for the 5xFAD mice and n = 8 for the ApoE^-/-^ mice. For three mice data could not be obtained because of their early removal from the study for humane reasons not related to the toxicity of the NP exposure. Combined with the genotyping verification, this resulted in the following animal numbers per group: ApoE^-/-^: control (n = 8); CeO_2_ (n = 7); 27% ZrO_2_-doped CeO_2_ (n = 8); 78% ZrO_2_-doped CeO_2_ (n = 8). 5xFAD: control (n = 16); CeO_2_ (n = 14); 27% ZrO_2_-doped CeO_2_ (n = 16); 78% ZrO_2_-doped CeO_2_ (n = 16). C57BL/6J: control (n = 10); CeO_2_ (n = 10); 27% ZrO_2_-doped CeO_2_ (n = 10); 78% ZrO_2_-doped CeO_2_ (n = 10). On day 52 and day 53 after the first exposure day behaviour tests were performed with the mice to assess for exposure-related neurotoxic effects. The animals were killed on day 57. The 4-week post-exposure period was included in the study design to address persistency of the effects and for the compromised mouse models to develop their respective disease phenotypes, i.e. Aβ formation in the brains of the 5xFAD mice and the atherosclerotic plaques in the ApoE^-/-^ mice.

### 2.3 Nanomaterial production, characterization and inhalation exposure

Production and detailed characterization of CeO_2_ NPs doped with different amounts of ZrO_2_ (ZrO_2_ contents in the doped NPs were 0 mol%, 27 mol% and 78 mol%) is described elsewhere (Dekkers et al. 2017). Approximately one week before the four-week exposure period, 20 samples of each NP (one for each day) were prepared at a concentration of 1 mg/mL from the stock dispersions (20, 20 or 29 mg/mL for 0%, 27% and 78% ZrO_2_-doped CeO_2_ NPs, respectively) by diluting with ultrapure water. Before use, stock and sample dispersions were sonicated for 5 min in an ultrasonic bath (Branson CPX2800, 40 kHz, 110W) before use to re-disperse any possible agglomerates. Aerosols of NPs were freshly generated using a spray nozzle technique, diluted with pressurized, clean and particle-free air, and heated to 24–25 °C (for detailed description see (Dekkers et al. 2017). Control animals were exposed to 3-h filtered air under the same exposure conditions (i.e. nose-only tubes) for the same amount of time. Prior to the day of exposure start all animals were trained to get used to the nose-only inhalation tubes.

### 2.4 Behaviour tests

At day 52 and 53 (i.e. 24 and 25 days after the last exposure day) the mice were examined by means of behavioural tests. At least 1 h before behavioural testing, mice were placed in the test room for acclimatisation. All tests were performed in dim red light. All test equipment and mazes were cleaned with 70% ethanol prior to each test to avoid odour recognition. On day 52, a string suspension test was performed: as a test of agility and grip strength (Miquel and Blasco, 1978), a 3 mm thick, 35 cm long cotton string was stretched between two escape platforms on top of two vertical poles. The mice were permitted to grasp the central part of the string by their forepaws, released immediately thereafter and allowed to escape to one of the platforms. A rating system from 0 to 7 was used during a single 60 s trial to assess each animals' performance (Moran et al. 1995) with the following modifications. Score: 0, unable to hang on the string; score 1, hangs only by forepaws; score 2, attempting to climb the string; score 3, climbing the string with four paws successfully; score 4, moving laterally along the string; score 5, escaping to the end of the string; score 6, falls while trying to climb the platform, score 7, reaches the platform.

On day 53, the X-maze task was performed to reflect activity and spatial working memory of mice by spontaneous alternation. Spontaneous alternation in rodents is based on the willingness to explore; a mouse tends to rotate in their entries between the four arms arranged in 90° position extending from a central space, which makes it more discriminative (arm sizes: 30 cm length, 8 cm width and 15 cm height). During 5 min test sessions, each mouse was placed in one arm and was allowed to move freely through the maze. The total number of arm entries was recorded using an infrared beam video camera during the 5 min interval to evaluate exploratory motor activity and this was then combined with the alternation to assess spatial working memory. Alternation was defined as successive entries into the four arms in overlapping quadruple sets (for example 1, 2, 3, 4 or 2, 3, 4, 1 but not 3, 2, 1, 3). Mice with impaired working memory will not remember visited arms leading to a decrease in spontaneous alternation (Holcomb et al. 1999). A successful entry was defined as a mouse entering one arm with all four paws. The alternation percentage was calculated as % of the actual alternations to the possible arm entries.

### 2.5 Necropsy and immunohistochemical analyses of paraffin embedded slices

At day 57, the mice were anesthetized with a mixture of ketamine and xylazine. The right brain hemispheres of 5xFAD mice were stored in 4% PFA for later processing for immunohistochemistry. The left brain hemispheres of C57BL/6J, ApoE^-/-^ and 5xFAD animals were rapidly dissected into cortex, olfactory bulb, cerebellum and midbrain. All brain regions were immediately transferred in liquid nitrogen and stored at −80 °C until further processing for Western blotting (see below). Dehydration was performed in a series of ethanol concentrations, followed by a transfer into xylene. Subsequently, the brains were embedded in paraffin. Four μm thick paraffin sections were cut using a sliding microtome and transferred on Superfrost Ultra Plus object slides (Thermo Scientific) and dried over night at 40 °C. Sections were deparaffinised in xylene, followed by rehydration in a series of ethanol (100%, 96%, 70%) and blocking of endogenous peroxidase by treatment with 0.3% H_2_O_2_ in PBS. Antigen retrieval was performed by boiling sections in 10 mM citrate buffer, pH 6.0 followed by incubation for 3 min in 88% formic acid. Non-specific antibody binding was blocked via incubation in 10% fetal calf serum (FCS) and 4% skimmed milk in 0.01 M PBS. Thereafter, slides were incubated overnight with primary anti human Aβ 42 antibody (clone G2-11, Cat.N0. MABN12, Merck Millipore, Darmstadt, Germany diluted 1:1000 in 0.01 M PBS and 10% FCS in a humid chamber at room temperature. After washing slices were incubated with a biotinylated anti-mouse secondary antibody (dilution 1:200 in 0.01M PBS and 10% FCS), and the signal was visualized avidin-biotin-complex-method (ABC) by a Vectastain kit (Vectorlabs, Burlingame, USA) using diaminobenzidine (DAB, Sigma-Aldrich, Deisenhofen, Germany)) as chromogen and Hematoxylin for nuclear counterstaining. Light microscope images from cortex and hippocampus were taken with 100x or 50x magnification, respectively, using a Zeiss Axiophot microscope equipped with AxioCam MRc (Carl Zeiss, Jena, Germany). Quantitative Aβ42 plaque analyses were performed via calculation of the percentage of total amyloid plaque load in the analysed area of the section. Plaque load was determined using ZEN 2011 image processing software (Zeiss) after a fixed adjustment of contrast threshold for stained Aβ42 plaques. Plaque load was interactively determined in the whole hippocampal area as well as in a defined cortex region. From each animal, three brain slides with an interspace of approximately 30 μm were analysed. For the immunostaining of ionized calcium-binding adapter molecule 1 (Iba-1), brain sections were incubated overnight with Iba-1 antibody (Cat No. GTX100042, GeneTex; dilution 1:1000 in 0.01 M PBS and 10% FCS) at 4 °C. The next day, slides were washed and incubated for 45 min at RT with biotinylated secondary antibody (dilution 1:200 in 0.01M PBS and 10% FCS. Staining was visualized using the ABC Vectastain kit (Vectorlabs, Burlingame, USA) and diaminobenzidine (DAB, Sigma-Aldrich, Deisenhofen, Germany)) as chromogen and Hematoxylin for nuclear counterstaining. Light microscope images from cortex and hippocampus were taken with 200x magnification using a Zeiss Axiophot microscope equipped with AxioCam MRc (Carl Zeiss, Jena, Germany). Iba-1 area (%) was quantified using ZEN 2011 image processing software (Zeiss) via calculation of the positive stained microglia (brown colour) in the defined cortical and hippocampal area.

### 2.6 Western blot analyses

Protein expression of ionized calcium-binding adapter molecule 1 (Iba-1), glial fibrillary acidic protein (GFAP), nuclear factor E2-related factor 2 (Nrf2) and heme oxygenase-1 (HO-1) was evaluated by Western blot to address whether the exposures to the CeO_2_ NPs resulted in neuroinflammation and oxidative stress. Iba-1 and GFAP represent well-established markers of activated microglia (Kovacs, 2017; Sasaki et al. 2001) and mature astrocytes in neuroinflammation (Li et al. 2020; Sofroniew and Vinters, 2010), respectively. The transcription factor Nrf2 is a master regulator of cellular responses to oxidants via its activation of oxidative stress response genes including HO-1. Both Nrf2 and HO-1 are implicated in neurotoxicity and neurodegenerative diseases including AD (Kanninen et al. 2009; Sandberg et al. 2014; Schipper et al. 2019). For the analysis of these markers, cortex brain tissues were homogenized in ~5 vol of ice-cold RIPA buffer for 2 h in a potter tissue grinder. The total protein level was evaluated with the BCA kit (Thermo) according to the manufactures protocol. Equal amounts of protein (50 μg) were loaded on a 4–12% precast NUPAGE gel (Invitrogen) and separated at 180 V in a Mini-PROTEAN II tank (BIO-RAD). The proteins were blotted at 250 mA for 45 min in a Mini Trans-Blot tank (BIO-RAD) on a 0.45 μm pore diameter nitrocellulose transfer membrane (Whatman, Schleicher & Schuell). With 5% milk in PBS-T (0.01 M PBS and 0.05% Tween-20) unspecific protein binding was blocked for 60 min. After the blocking, the membrane was incubated with the primary antibody: GFAP (Cat No. ab7260, Abcam, 1:5000), Iba-1 (Cat No. GTX100042, Gentex, 1:1000), HO-1 (Cat No. AB1284, Merck, 1:1000), Nrf2 (C-20) (Cat No. sc-722, Santa Cruz,1:500) overnight at 4 °C. Next day, secondary hrp-conjugated antibody and β-Actin-hrp (AC-15) (Cat No. A384, Sigma,1:50000) was incubated for 1 h at room temperature. Detection of proteins was performed with ECL solution (GE Healthcare) and visualized with CHEMI Premium Imager (VWR). With the use of ImageJ software (National Institutes of Health, Bethesda, USA) quantification of protein expression was evaluated relative to β-actin protein level.

### 2.7 Statistical analyses

Data were analysed using IBM-SPSS (version 22) and are expressed as mean ± SEM unless stated otherwise. Data were evaluated by one-way analysis of variance (ANOVA) with Dunnett post-hoc analysis using the air exposed animals as statistical control group. Differences were considered statistically significant at p < 0.05.

## 3 Results

### 3.1 Exposure conditions

Detailed characteristics of NPs and their particle size distributions, mass and number exposure concentrations as well as lung deposited dose estimations for the inhalations are described in detail elsewhere (Dekkers et al. 2017, 2018). Briefly, the different CeO_2_ particles had a primary particle size of 4.7 ± 1.4 nm. The gravimetric mass concentrations and size distribution of the aerosols were almost identical for the exposures to the CeO_2_, 27% Zr-doped CeO_2_ and 78% Zr-doped CeO_2_ NPs.

### 3.2 Effects on motor activity and cognitive function

**Fig. 1:**
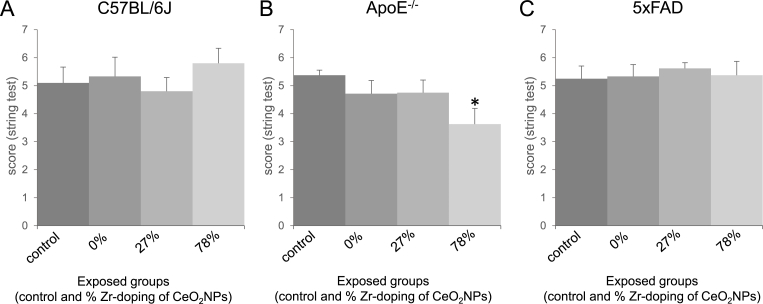
***Effects of redox-modified* CeO**_**2**_***on performance in the string suspension task****.* Female C57BL/6J (A), ApoE^-/-^ (B) and 5xFAD (C) mice were exposed to clean air (control) or CeO_2_ and 27% ZrO_2_-doped CeO_2_ or 78% ZrO_2_-doped CeO_2_ NPs via inhalation. The ability of the mice to escape to a platform within 60 s was measured and transferred to a rating system from 0 to 7 whereby a higher score represents a better performance. Data are expressed in mean ± SEM, *statistical significantly different from the respective control in Dunnet post-hoc test following one-way ANOVA with p < 0.05. Number of animals per group: ApoE^-/-^: control (n = 8); CeO_2_ (n = 7); 27% ZrO_2_-doped CeO_2_ (n = 8); 78% ZrO_2_-doped CeO_2_ (n = 8). 5xFAD: control (n = 16); CeO_2_ (n = 14); 27% ZrO_2_-doped CeO_2_ (n = 16); 78% ZrO_2_-doped CeO_2_ (n = 16). C57BL/6J: control (n = 10); CeO_2_ (n = 10); 27% ZrO_2_-doped CeO_2_ (n = 10); 78% ZrO_2_-doped CeO_2_ (n = 10).

The effect of exposure to redox-modified CeO_2_ NPs on the behaviour of the mice was determined using the string suspension test and the X-maze test. The string suspension task was used to assess for motor activity, where mice were allowed to grasp a cotton string stretched between two vertical poles and the ability of the animals to cling on and move to one of the platforms on top of the poles within 60 s was measured and scored. Results are shown in Fig. 1.

There was no significant difference in test performance between the controls (clean air exposed mice) of the three different strains. Exposure of the mice with the distinct CeO_2_ NPs did not affect the performance of the C57BL/6J mice and the 5xFAD transgenic mice in the string suspension task. However, among the ApoE^-/-^ mice the string suspension test performance was diminished in the group that was exposed to the 78% Zr-doped CeO_2_ NPs compared to controls, indicative of an adverse impact on the motor function (Fig. 1). The inhalation exposures to the CeO_2_ NPs that contained less (27%) or no (0%) Zr did not significantly alter the behaviour of the ApoE^-/-^ mice in the string suspension test in comparison to the clean air exposed animals.

**Fig. 2:**
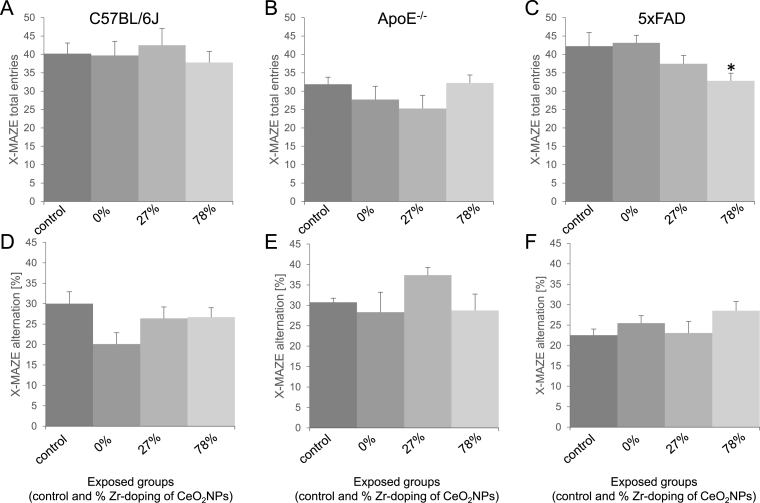
***Effects of redox-modified CeO2 on performance in the X-maze task.*** Female C57BL/6J (A, D), ApoE^-/-^ (B, E) and 5xFAD (C, F) mice were exposed to clean air (control) or CeO_2_ and 27% ZrO_2_-doped CeO_2_ or 78% ZrO_2_-doped CeO_2_ NPs via inhalation. After this treatment, the differently exposed groups were subjected to the X-maze task. Mice were place in the maze for 5 min. The behavioural parameters analysed were total arm entries (A, B, C) and alternation (D, E, F) and expressed in mean ± SEM. *Statistical significance different from the respective control in Dunnet post-hoc test following one-way ANOVA with p < 0.05. Number of animals per group: ApoE^-/-^: control (n = 8); CeO_2_ (n = 7); 27% ZrO_2_-doped CeO_2_ (n = 8); 78% ZrO_2_-doped CeO_2_ (n = 8). 5xFAD: control (n = 16); CeO2 (n = 15); 27% ZrO_2_-doped CeO_2_ (n = 16); 78% ZrO_2_-doped CeO_2_ (n = 16). C57BL/6J: control (n = 10); CeO_2_ (n = 10); 27% ZrO_2_-doped CeO_2_ (n = 10); 78% ZrO_2_-doped CeO_2_ (n = 10).

The X-maze task was performed to assess for locomotor activity and spatial working memory of the mice in relation to the different inhalation exposures. The results of these investigations are shown in Fig. 2. In contrast to the string suspension test, for this task some differences were already noted between the controls (clean air) of the different mouse models. On the one hand, for the ApoE^-/-^ control group the total number of arm entries tended to be lower than for the C57BL/6J and 5xFAD controls. On the other hand, the alternation (%) in the test tended to be lower for the 5xFAD control mice in comparison to the C57BL/6J and ApoE^-/-^ controls. However, in both cases the observed differences were not statistically significant.

In concordance with the string suspension task, the X-maze test also revealed a significant effect on behaviour following inhalation exposure to the 78% Zr-doped CeO_2_ NPs, whereas the other types of NPs showed no effects. In this case, however, the effect was seen in the 5xFAD mouse model: The 5xFAD mice that had been exposed to the 78% Zr-doped CeO_2_ NPs showed a significantly reduced number of total arm entries compared to the control 5xFAD mice, indicative of a decreased exploratory motor activity for this treatment group (Fig. 2). However, the alternation in the X-maze task, which is an indicator of the spatial working memory of mice, did not differ between these groups. In fact, the alternation percentage among the 5xFAD groups tended to be highest in the 78% Zr-doped CeO_2_ NPs. In the ApoE^-/-^ and C57BL/6J mice, no significant treatment-related effects on locomotor activity and spatial working memory were found with the X-maze testing.

### 3.3 Effects on Aβ plaque formation and markers of neuroinflammation and oxidative stress

**Fig. 3:**
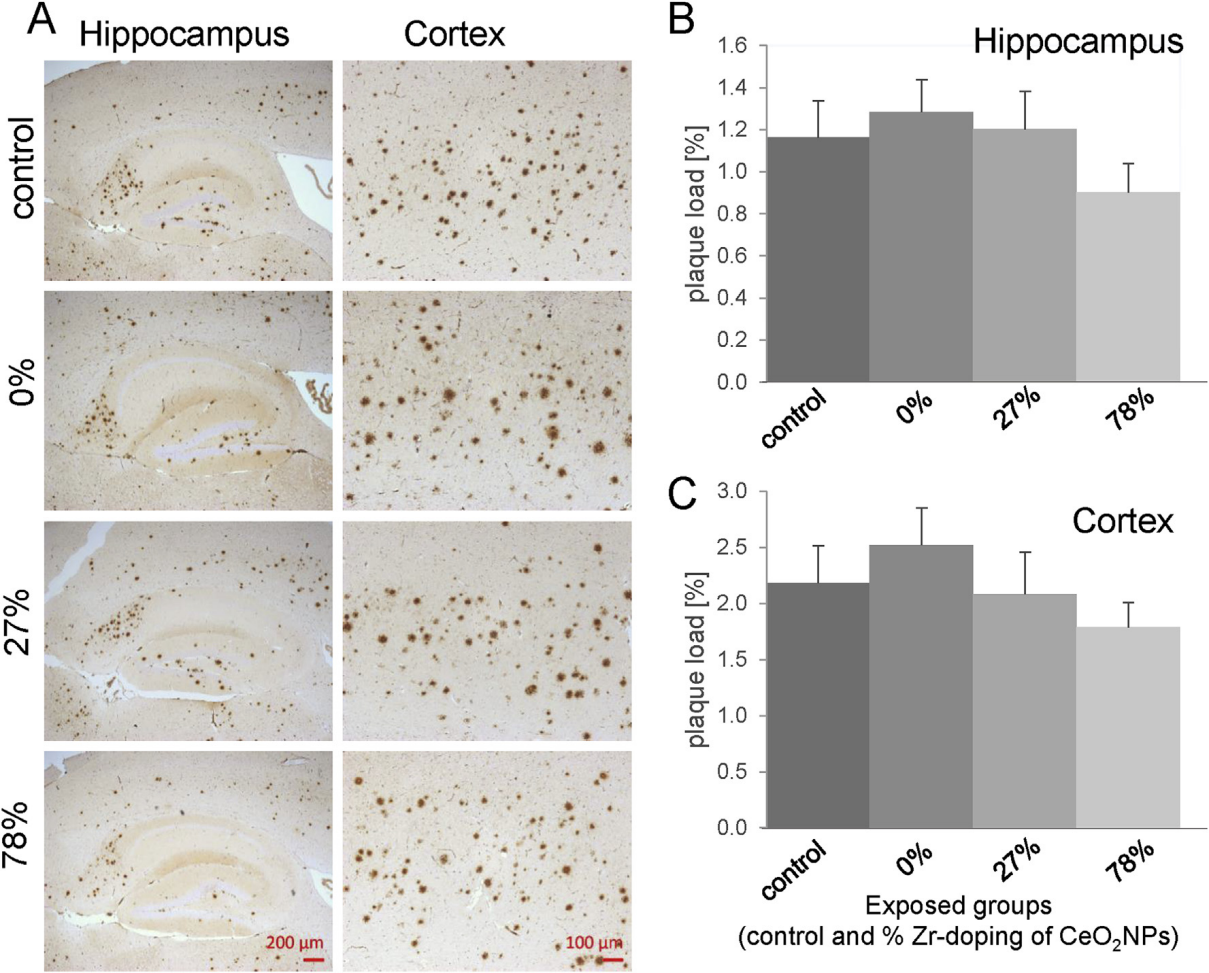
***Effect of redox-modified* CeO**_**2**_***NPs inhalation on β-Amyloid pathology in 5xFAD mice****.* Aβ plaque load was determined in parasagittal brain slices of 5xFAD mice after exposure to clean air (control n = 16), CeO_2_ (n = 15), 27% ZrO_2_-doped CeO_2_ (n = 16) or 78% ZrO_2_-doped CeO_2_ (n = 16) NPs. Aβ42 was visualized by IHC in 4 μm sections of paraffin-embedded brain hemispheres (Representative pictures are shown in A). For quantification, plaque load was determined in the hippocampus (B) and in the cortex (C) using image analysis software and calculated as the percentage area occupied by Aβ immunostaining expressed in mean ± SEM. For determination of plaques in the cortex, whole image sections were evaluated while the hippocampus regions were defined by hand to evaluate only the hippocampus. A trend was observed of reduced Aβ plaques in the brains of mice exposed to the 78% Zr-doped CeO_2_ NPs, but this effect was not statistically significant.

**Fig. 4:**
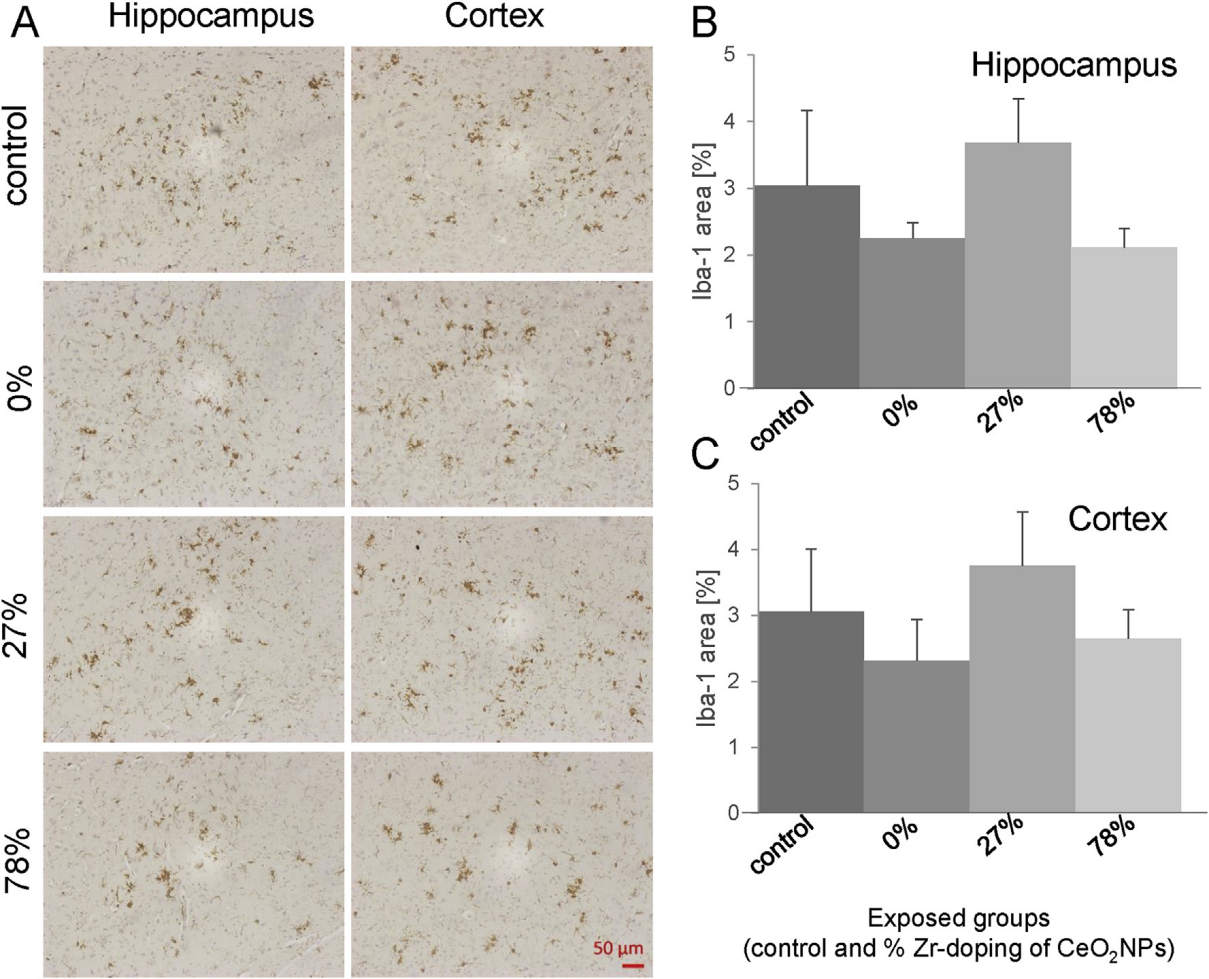
***Effect of redox-modified* CeO**_**2**_***NP inhalation on Iba-1 immunostaining in hippocampus and cortex of 5xFAD mice***. Parasagittal brain slices of 5xFAD mice exposed to clean air of CeO_2_ NPs with different doping of Zr (n = 6 per group), were stained with an antibody against Iba-1 to detect activated microglia (representative pictures are shown in A). For quantification, Iba-1 stain was determined in (B) CA1/subiculum of the hippocampus (200-fold microscopic magnification) and (C) cortex layer 5 (200-fold microscopic magnification) using image analysis software and calculated as the percentage area occupied by Iba-1 immunostaining and expressed in mean ± SEM.

Histopathologically, AD is characterized by the presence of extracellular senile plaques and intracellular neurofibrillary tangles (Beyreuther and Masters, 1991; Selkoe, 2001). To investigate the impact of the exposure to different redox-modified CeO_2_ NPs on the level of Aβ plaque formation, parasagittal brain slices of the 5xFAD mice were stained with an antibody against human Aβ42, and the Aβ plaque load was determined in hippocampus and cortex. The results of this analysis are shown in Fig. 3. There were no significant differences in plaque formation between the different treatment groups: the inhalation exposures to NPs, irrespective of their redox modification, did not result in an acceleration of the Aβ plaque formation in this transgenic mouse model of AD. To determine whether the inhalation of redox-modified CeO_2_ NPs affect the level of neuroinflammation in the brains of the 5xFAD mice, the amount of Iba-1 positive microglia cells was assessed in the same brain regions using immunohistochemical analysis. Compared to the clean air exposed 5xFAD mice, the number of activated Iba-1 positive microglia cells was not significantly altered in the brain of NPs treated 5xFAD mice (Fig. 4).

**Fig. 5:**
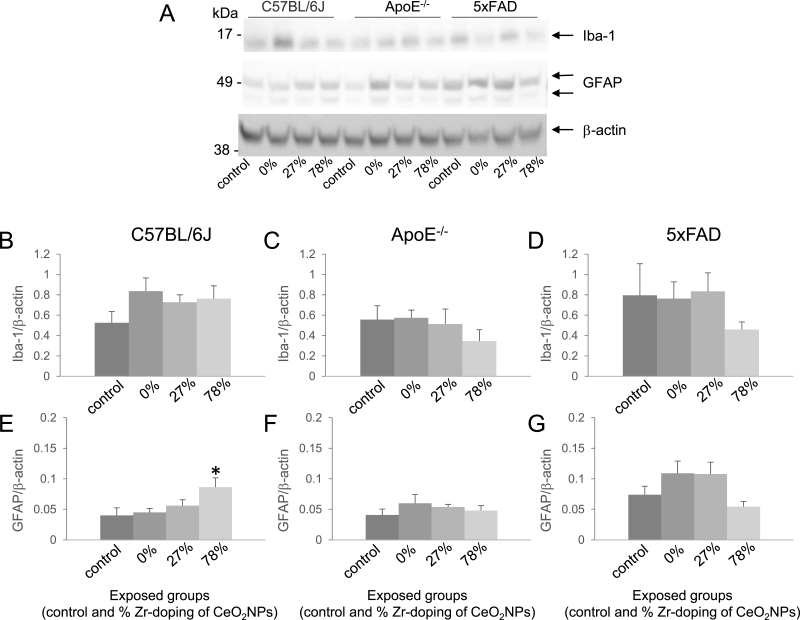
***Effect of redox-modified* CeO**_**2**_***NP inhalation on Iba-1 and GFAP protein levels***. Levels of Iba-1 (B, C, D) and GFAP (E, F, G) were assessed by Western blot analysis in lysates of the cortex of female C57BL/6J (B, E), ApoE^-/-^ (C, F) and 5xFAD (D, G) exposed to clean air or CeO_2_ NPs with different doping of Zr (representative blots are shown in A). Data were normalized to the level of β-actin and expressed in mean ± SEM. * Statistical significance different from the respective control in Dunnet post-hoc test following one-way ANOVA with p < 0.05. Number of animals per group: ApoE^-/-^: control (n = 5); CeO_2_ (n = 5); 27% ZrO_2_-doped CeO_2_ (n = 5); 78% ZrO_2_-doped CeO_2_ (n = 4). 5xFAD: control (n = 4); CeO_2_ (n = 4); 27% ZrO_2_-doped CeO_2_ (n = 4); 78% ZrO_2_-doped CeO_2_ (n = 4). C57BL/6J: control (n = 5); CeO_2_ (n = 5); 27% ZrO_2_-doped CeO_2_ (n = 5); 78% ZrO_2_-doped CeO_2_ (n = 5).

To further evaluate the potential effect of the different redox-modified CeO_2_ NPs on neuroinflammation and oxidative stress, cortical brain tissues of all three mouse models were analysed by Western blotting for the expression of Iba-1 and GFAP as well as Nrf2 and HO-1, respectively. Results for Iba-1 and GFAP are shown in Fig. 5. As can be seen in the figure, the Western blot analyses for Iba-1 confirmed the absence of treatment-related changes in abundance of Iba-1 positive microglia cells in the 5xFAD mice by immunohistochemistry: No significant differences Iba-1 protein levels were detected in the 5xFAD mice in association with the CeO_2_ inhalation exposures. Likewise, the protein levels of Iba-1 were not significantly altered in the brains of the C57BL/6J and ApoE^-/-^ mice. Next, the protein level of the astrocyte marker GFAP was analysed. In the ApoE^-/-^ and 5xFAD mice, no treatment related effects on GFAP protein level could be observed. Interestingly, however, the C57BL/6J mice exposed to the 78% Zr-doped CeO_2_ NPs displayed significant higher GFAP levels whereas inhalation of both other types of NPs showed no effect.

**Fig. 6:**
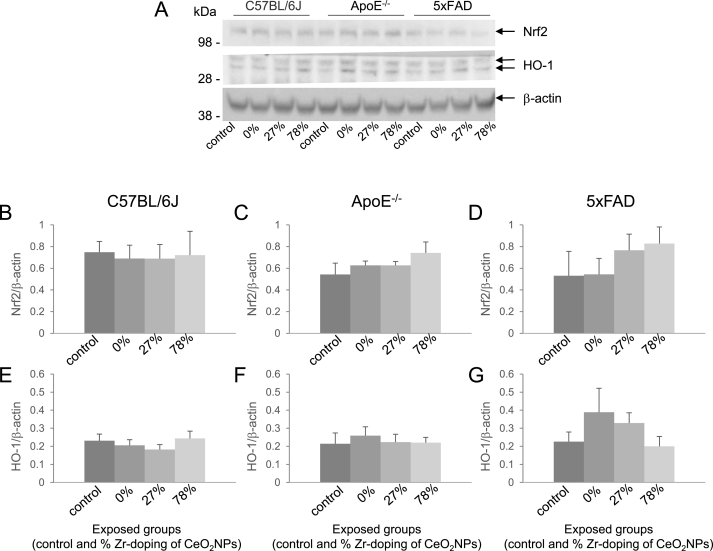
***Effect of redox-modified* CeO**_**2**_***NP inhalation on Nrf2 and HO-1 protein levels***. Lysates of the cortex of C57BL/6J (B, E), ApoE^-/-^ (C, F) and 5xFAD (D, G) mice exposed to clean air or CeO_2_ NPs with different doping of Zr were subjected to Western blot analysis. Levels of Nrf2 (B, C, D) and HO-1 (E, F, G) were normalized to the level of β-actin and expressed in mean ± SEM. Representative blots are shown in A. Number of animals per group: ApoE^-/-^: control (n = 5); CeO_2_ (n = 5); 27% ZrO_2_-doped CeO_2_ (n = 5); 78% ZrO_2_-doped CeO_2_ (n = 4). 5xFAD: control (n = 4); CeO_2_ (n = 4); 27% ZrO_2_-doped CeO_2_ (n = 4); 78% ZrO_2_-doped CeO_2_ (n = 4). C57BL/6J: control (n = 5); CeO_2_ (n = 5); 27% ZrO_2_-doped CeO_2_ (n = 5); 78% ZrO_2_-doped CeO_2_ (n = 5).

The effect of exposure to redox-modified CeO_2_ NPs on the protein expressions of Nrf2 and its downstream target HO-1 are shown in Fig. 6. Inhalation exposure to the NPs, irrespective of their redox-modification, did not affect the level of Nrf2 or HO-1 in the brains of C57BL/6J, ApoE^-/-^ and 5xFAD mice (Fig. 6). In the 5xFAD mice, a tendency of increasing Nrf2 protein levels with increased Zr-doping was noted suggestive of increasing oxidative stress response. However, the observed differences were not statistically significant and were also not further substantiated by the HO-1 findings for the same mice.

## 4 Discussion

The experiments performed in this study formed part of a large study to assess the influence of redox activity on the toxicity of inhaled CeO_2_ NPs in mice, by comparison of the effects of different quantities of Zr-doping. Detailed physicochemical and exposure characteristics of the NPs as well as the pulmonary and cardiovascular findings in the exposed mice have been published in a separate paper (Dekkers et al. 2017). In all three mouse models (C57BL/6J, 5xFAD, ApoE^-/-^) the four-week inhalation exposures were without any major toxicological effects in the lungs. In the ApoE^-/-^ mouse model of vascular disease, the inhalation exposures to the NPs did not cause a statistically significant change in the overall size of atherosclerotic plaques. However, there was a trend towards an increased inflammatory cell content (i.e. macrophage-derived foam cells) in the plaques with the inhalation of CeO_2_ NPs with increasing ZrO_2_ content (Dekkers et al. 2017).

In the present study, we evaluated whether inhalation exposure to these NPs could also cause neurotoxicity and promote AD. Therefore, mouse behaviour tests were performed in all three mouse models to explore effects on motor activity and cognitive function. Brain tissue protein levels of HO-1, Nrf2, Iba-1 and GFAP were measured to address the role of oxidative stress and neuroinflammation. The potential effects of the inhaled CeO_2_ NPs on amyloid-β plaque formation were assed in the 5xFAD mouse model. In this study, we could observe specific effects that were dependent on the mouse model as well as the NP modification. While the behaviour effects were observed in both compromised mouse models, increased protein levels of GFAP were found only in the healthy C57BL/6J mice. These significant effects were observed exclusively for the CeO_2_ NPs that were doped with the highest amount of Zr (78%). In the ApoE^-/-^ mice, the four-week inhalation exposure to these specific NPs resulted in a significantly diminished performance in the string suspension test. Such effect could be an indication of a greater susceptibility to an impaired forced motor performance in this mouse model of vascular disease. In the 5xFAD mice, the exposure to the 78% Zr-doped CeO_2_ NPs resulted in a significant reduction of the total of arm entries in the X-maze task. This latter effect suggests a possible reduction in explorative locomotor activity for this mouse model of AD. However, alternation behaviour in the X-maze test, which is an indicator of cognitive performance, was not impaired in the same treatment group.

Interestingly, while the motor performance effects on behaviour were observed with the two disease models, no behavioural effects were seen in the healthy (C57BL/6J) mice. Rodent models of susceptibility and disease are being increasingly used in toxicological studies exploring air pollution to better understand the underlying mechanisms (Oberdorster et al., 2005; Stone et al. 2017). In line with our present findings with the Zr-doped CeO_2_ NPs, impaired motor performance was observed following diesel engine exhaust inhalation exposure in 5xFAD mice but not in their wildtype littermates (Hullmann et al. 2017). Studies with diesel exhaust particles in high-fat fed ApoE deficient mice have also demonstrated the value of this susceptibility model over wildtype mice to support the epidemiological evidence that links exposure to airborne particles to cardiovascular disease (Miller et al. 2013). Interestingly, the behaviour changes in the two compromised mouse models were exclusively seen with the highest Zr-doped CeO_2_, indicating that these effects appear to depend on the redox-activity of the inhaled NPs. The introduction of Zr into the crystalline structure of CeO_2_ NPs is considered to enhance their antioxidant properties (Tsai et al. 2008). As such, one would have expected a possibly protective effect for the undoped CeO_2_ NPs. However, our present findings are in line with the previously reported effects of the inhalation exposures on the inflammatory content of atherosclerotic plaques in the ApoE^-/-^ mice, which revealed an increased presence of macrophage-derived foam cells for CeO_2_ NPs with increasing Zr content (Dekkers et al. 2017).

Behaviour tests form an important component of neurotoxicity testing (Moser, 2011; OECD, 1997). It is therefore tempting to speculate that the observed motor performance effects in the ApoE^-/-^ and 5xFAD mice result from a direct neurotoxic effect of the high Zr-doped CeO_2_ NPs. Indeed, several studies that have explored the pulmonary toxicity of NPs, including CeO_2_, indicate that their adverse effects are driven by oxidative stress and inflammation (Unfried et al. 2008; Morimoto et al. 2016; Stone et al. 2017; Schwotzer et al. 2018). However, in our present inhalation study we found no significant treatment related changes in HO-1 or Nrf2 for all three mouse models. In contrast, Hardas and colleagues observed increased HO-1 in rat brain upon intravenous administration of CeO_2_ NPs (Hardas et al. 2014). The fundamental differences in exposure route and dose offer a plausible explanation for these contrasts. The brains of ApoE^-/-^ and 5xFAD mice in our inhalation study also did not display significant treatment related changes in protein levels of Iba-1 and GFAP, even for the groups that were exposed to the 78% Zr-doped CeO_2_ NPs. Taken together, this suggests that the motor function effects which we observed in both compromised mouse models were not mediated by local oxidative stress and neuroinflammation.

Surprisingly, however, increased protein levels of GFAP were observed in the cortex of the healthy C57BL/6J mice, the only mouse model that did not show significant changes in (motor function) behaviour. On the one hand, this adds further support to the absence of a mechanistic link between neuroinflammation and motor activity changes for inhaled CeO_2_ NPs. On the other hand, the finding again indicates the importance of the redox-properties of CeO_2_ NPs, as the effect on GFAP was only seen with the particles that were doped with the highest amount of Zr (78%). Increased GFAP levels were previously also found in rat brain following repeated inhalation exposures to steel welding fumes (Antonini et al. 2009). In contrast to our findings, increased GFAP levels were observed in ApoE^-/-^ mice after long term inhalation of ambient ultrafine particles (Kleinman et al. 2008). In another study with in C57BL/6 mice, the long term inhalation of fine (μm size mode) ambient particulate matter (PM_2.5_) did not cause significant changes in brain levels of GFAP and Iba-1 (Bhatt et al. 2015).

The ApoE^-/-^ mice were selected *a priori* for the investigation of cardiovascular effects following NP inhalation exposure, however, due to the logistical requirements of the extensive tissue collection, we were unable the further evaluate the brain tissue from these mice by immunohistochemistry (Dekkers et al. 2017). However, the brains of the 5xFAD mice were prioritised to address the potential impact of (undoped and Zr-doped) CeO_2_ NPs on the development of the neurodegenerative processes. Previously, we demonstrated an accelerated amyloid plaque load formation (whole brain Aβ42 protein levels) in 15 week old female 5xFAD mice following a three-week diesel engine exhaust inhalation exposure (0.95 mg/m^3^, 6 h/day, 5 days/week) (Hullmann et al. 2017). In the present study, however, we did not observe a significant alteration in the β-amyloid pathology in the brains of 5xFAD animals following four-week inhalation exposure to the (Zr-doped) CeO_2_ NPs (4 mg/m^3^ for 3 h/day, 5 days/week). Moreover, in alignment with the Western blot findings, the brains of the 5xFAD mice did not reveal significant differences in immunostaining of Iba-1. Combined with the observed absence of (cognitive) behaviour changes in the 5xFAD mice, these data argue against the hypothesis that CeO_2_ NPs may promote AD pathology in association with their redox-activity. The motor performance changes observed to NPs in the ApoE^-/-^ and 5xFAD mice may not necessarily be related to a direct neurotoxic effect in isolation, but instead due to indirect effects, or alternatively, a result of an interaction between the exposure and increased susceptibility of both disease models. Age related changes in motor performance are well-described in the 5xFAD mouse model (Jawhar et al. 2012; O'Leary et al. 2018) and have also been reported for the ApoE^-/-^ mice (Raber et al. 2000; Zerbi et al. 2014). Importantly, however, we did not observe statistically significant differences in behaviour test performance of the (clean air exposed) control mice between the three different mouse modes. This indicates that there was no major behaviour impairment *per se* in the two mouse disease models, and also suggests that it is unlikely that the effects of NPs on ApoE^-/-^ mice were principally due to the high-fat diet fed to the mice. Further research is needed to verify the potential adverse impact of inhaled CeO_2_ NPs on motor function and to unravel the mechanism that could explain the redox-involvement for these metal oxide NPs.

Up to now, there is only very limited data about the potential neurotoxic effects of CeO_2_ NPs in association with inhalation exposure. A recent study showed that female ICR mice exposed to CeO_2_ particles (intranasal instillation, daily dose of 40 mg/kg body weight) of varying sizes (i.e. 35 nm, 300 nm and >1 μm) displayed significantly increased GFAP expression in the hippocampus and olfactory bulb. The authors claim that intranasal instillation of CeO_2_ particles induced damage within the olfactory bulb and hippocampus, but that particle size does not play a major role in the observed adverse responses (Liu et al. 2016). Nemmar and colleagues reported increased levels of the inflammatory cytokine Tumor Necrosis Factor-α, reactive oxygen species and DNA damage in the brains of mice by CeO_2_ NPs, 24 h after a single intratracheal instillation (0.5 mg/kg) (Nemmar et al. 2017). However, for the aforementioned studies the observed effects require perspective on the method and site of administration in the respiratory tract for the CeO_2_ particles, when compared to the outcomes of our present controlled inhalation exposure study. This relates to the obvious differences in dose and dose-rate of the NPs (i.e. bolus application *versus* inhalation) as well as to the regional deposition in the respiratory tract organ (i.e. nasal *versus* alveolar).

## 5 Conclusions

We have investigated the neurological effects of redox-modified CeO_2_ NPs using varying levels of Zr-doping (0%, 27% and 78%), after four-week inhalation exposures in three different mouse models. Our study findings reveal that the subacute inhalation exposure to CeO_2_ NPs did not cause major cognitive behavioural impairments in mice or promote amyloid-β plaque formation and neuroinflammation in the 5xFAD transgenic mouse model of AD. However, motor performance changes were observed both in the 5xFAD and ApoE^-/-^ mice for the CeO_2_ NPs that were doped with the highest amount of Zr. In healthy C57BL/6J mice, the same particles caused increased GFAP levels in the absence of behaviour changes. The observed behavioural effects in the two compromised models were not substantiated further by changes in markers of neuroinflammation and oxidative stress. Therefore, further investigations are warranted to unravel the mechanism whereby inhaled CeO_2_ NPs can affect motor activity in a redox activity dependent manner.

## Funding

The work leading to these results has received funding from the European Union Seventh Framework Programme for research, technology development and demonstration [grant agreement no. 310451 (NanoMILE)] and the Netherlands Food and Consumer Product Safety Authority (NVWA) [V090016]. MRM is supported by the 10.13039/501100000274British Heart Foundation [SP/15/8/31575; CH/09/002].

## Declaration of competing interest

The authors declare that they have no competing interests.
